# Musculoskeletal Health Effects of Manual Sandcrete Block Handling Among Construction Artisans: Implications for Industry Practice and Training

**DOI:** 10.3390/ijerph22111689

**Published:** 2025-11-07

**Authors:** Kofi Owusu Adjei, Murendeni Liphadzi, Francis Kwesi Bondinuba, Cecilia Modupe Mewomo, Haruna Domanamwin Abudu

**Affiliations:** 1 Department of Construction Management and Quantity Surveying, University of Johannesburg, P.O. Box 524, Auckland Park 2006, South Africa; 201494604@student.uj.ac.za (K.O.A.); mliphadzi@uj.ac.za (M.L.); 2 Department of Construction Technology and Quantity Surveying, Faculty of Built and Natural Environment, Kumasi Technical University, P.O. Box 854, Kumasi 00233, Ghana; francis.kbondinuba@kstu.edu.gh; 3 Faculty of Engineering and the Built Environment, Durban University of Technology, P.O. Box 1334, Durban 4000, South Africa; 4 The Urban Institute, School of Energy, Geoscience, Infrastructure and Society, Heriot-Watt University, Edinburgh EH14 5AS, UK; 5 Department of Engineering Technology, Tarleton State University, Stephenville, TX 76402, USA; mmewomo@tarleton.edu; 6 Department of Construction Management and Quantity Surveying, Faculty of Engineering and the Built Environment, Durban University of Technology, P.O. Box 1334, Durban 4000, South Africa

**Keywords:** artisans, health and well-being, health and safety, manual work, musculoskeletal, sandcrete block

## Abstract

This study examines the occupational health challenges faced by artisans in Ghana’s Ashanti Region. The study employed a purposive sampling technique to select 425 masons through questionnaires. The data collected included workers’ mass, block weights, and health problems. The analysis identified fourteen health problems, with headaches, muscle pain, lower back pain, chronic pain, and bruises being most prevalent. Artisan weight negatively correlated with weights of 125 mm blocks (r = −0.202, *p* < 0.01) and 150 mm blocks (r = −0.248, *p* < 0.01). Additionally, artisan weight showed a negative correlation with working hours (coefficient = −0.133, *p* < 0.05), as did body weight with resting hours (coefficient = −0.217, *p* < 0.05). Higher educational qualifications correlated positively with lifting-related health issues (coefficient = 0.259, *p =* 0.000) and negatively with weather-related issues (coefficient = −0.154, *p =* 0.002). Moreover, the number of working days per week was positively correlated with lifting problems (coefficient = 0.270, *p* < 0.05). The study recommends redesigning block sizes to reduce physical strain. Additionally, policy measures such as reducing machinery import taxes are suggested to encourage mechanisation. The study further emphasises the need for training institutions to incorporate occupational health education into artisan training programs.

## 1. Introduction

The construction industry encompasses numerous global professional occupations that specialise in infrastructure projects. It is an industry with a high risk of occupational injuries because the cause of workplace injury or illness is multifaceted [1]. The construction industry is more susceptible to injuries and illnesses than other industries. Some authors have found that the risk of death in construction is greater compared to the rate in other industries [2]. The construction sector is vital to the economy because it produces goods and services such as infrastructure, electricity lines, water supplies, and roads. Unemployed and unskilled workers can find work in this industry in developing nations with high unemployment rates, such as Ghana. Over 600,000 people are employed in the construction industry, making up roughly 7% of the working population, according to the Ghana Statistical Service’s 2015 labour force report. Nonetheless, the figure might be greater because of unrecorded informal labourers [3]. Considering the high frequency of fatal occupational injuries, the construction sector is among the largest hazardous sectors in the world [4].

Compared to other industries, the construction industry’s productivity is low due to its many challenges, which impede growth. Stakeholders recognise the construction industry’s historical resistance to change and emphasise the necessity of greater digitisation and automation in the field [5]. The ability to operate robots remotely and intelligent construction is a rapidly emerging technology predicted to enhance worker health. Because of its physical characteristics, this technology effectively reduces the damage caused by noise and dust [6]. Low back injuries in the construction industry are on the rise, even with improvements in automation and material handling equipment [7]. Despite notable advancements in safety management techniques, there are still many workplace mishaps in the construction sector that result in injuries to various body parts of construction workers [8].

Skilled and semi-skilled labour, such as carpenters, masons, painters and steel benders, are crucial for building structures in the African diaspora. Despite lacking professional training, their role is essential for the success of construction projects, filling the void in the sector. Their involvement is crucial for the success of any construction project [8,9]. Ghana’s low-tech construction sector, heavily reliant on labour-intensive techniques, poses a high risk of occupational health and safety incidents and injuries [4]. The World Health Organisation (WHO) reports that construction workers are more susceptible to occupational diseases and illnesses due to exposure to health hazards such as dust and noise, resulting in significant financial losses for families and society [6].

The health implications of these artisans in the construction industry are becoming very alarming in both developed and developing countries. Over 20 years (1/2000–8/2020), the United States construction industry documented 23,057 fall accidents, according to data from the Occupational Safety and Health Administration (OSHA) statistics [1]. Occupational safety and health (OSH) are significant issues in developing countries, particularly for informal workers such as the masons. However, there is limited information on the artisan mason’s health status, which is generally poor [10].

Previous studies demonstrating the body position in sandcrete blockwork include Ryu et al. [11] and Tao et al. [12]. However, in a developing nation like Ghana, the health issues these manual workers face have not been examined, and as a result, the risks to their health have worsened. In Ghana, manual construction includes lifting, lowering, pushing, pulling, carrying, and handling construction units. The most prevalent ways that artisans operate with standardised blocks when executing manual construction tasks include frequent bending, twisting, impulsive motions, and bent-over postures. Moreover, the weight of blocks and the health problems posed by the masons have not gained attention in the literature; for that matter, a clear view of the impact of building units on the health of artisans who manually use them in construction needs to be investigated. Thus, the study examines the health of artisans in the construction industry who use standardised blocks.

Purpose of the Study: Thus, this study aims to assess the musculoskeletal health effects of manual sandcrete block handling among construction artisans in Ghana. It specifically seeks to examine the types of musculoskeletal disorders (MSDs) reported, the handling practices contributing to these conditions, and the level of awareness of health risks among these workers. The study further explores the implications of these findings for construction industry practice and artisan training.

Research Questions

1. What types of musculoskeletal health issues are commonly experienced by construction artisans engaged in manual sandcrete block handling?
2. What specific manual handling practices contribute to these musculoskeletal health issues?
3. How do artisan weight and block weight affect musculoskeletal disorders among construction artisans handling sandcrete blocks?

The study’s structure begins with the Introduction, which outlines the background, objectives, and relevance of occupational health among construction artisans using standardised blocks. A brief Literature Review highlights key issues and existing gaps. The Methodology section describes the research design, data collection, and analysis procedures. The Results section presents the main findings, followed by the Discussion, which interprets these findings in relation to existing literature, including the roles of artisans and the associated health risks. The study concludes with the Conclusions, summarising key insights and offering relevant recommendations.

## 2. Literature Review

### 2.1. Overview of Construction Artisans

Artisans are skilled technicians who work on-site and collaborate with other professionals. They have acquired various skills through jobs or skill acquisition centres. Artisans in the building construction industry include masons, carpenters, rebar benders, tillers, and plumbers. The success of construction projects relies on the skills and commitment of artisans despite the labour-intensive and dangerous nature of construction activities [13]. Most craftspeople receive informal apprenticeship training through which they acquire creative and entrepreneurial abilities. Learning a trade through an internship under a master’s supervision is called apprenticeship training. It includes all agreements where young people develop bonds with a master who agrees to teach them the trade in exchange for a set amount and time of service [14].

Craftsmen are considered crucial in the construction process, as per construction jargon [9]. In the context of traditional construction, artisans are people trained in both art and trade to perform specialised technical tasks. These tasks include carpentry, joinery, iron welding, masonry, bricklaying, painting, decoration, and earthwork. Artisans are trained in their craft as a profession to earn a source of income. Artisans are individuals who teach and impart the fundamental principles of a particular craft [9]. Their inputs significantly impact the final product’s time, cost, and quality. Proper management and motivation are crucial for survival and sustainable productivity improvement [2]. Different schools of thought have diverse ideas about categorising artisans based on their domain performance. For example, one school of thought defined an artisan mason as someone who performs moulding and laying of bricks or blocks for facilities, while other schools of thought defined a mason or bricklayer as someone who uses stone works to decorate building façades and cast concrete works [9].

It is well known that artisans and craftsmen have specialised abilities for making things by hand. They are extraordinarily talented people who can create things out of basic elements. Most people in developing nations are employed in the construction sector, although the majority of them are impoverished. Before establishing their trades, most of these craftsmen or artisans complete an apprenticeship. Most Ghanaian artisans are small-scale and lack entrepreneurial skills, with few completing vocational or tertiary education, and a significant number not pursuing entrepreneurial ventures [15]. Tradespeople, who typically receive training through the traditional apprenticeship system, are often ranked lowest in the labour market. Artisans, despite their crucial role in skill development and economic growth, often lack formal training, making them unprepared to adapt to modern technological advances [14].

### 2.2. Occupational Health Risks Faced by Artisans in Standardised Block Usage

Over the past two decades, the sandcrete block manufacturing industry in Ghana and other parts of sub-Saharan Africa has undergone a significant transformation. Advances in production techniques, including the use of better-quality cement, improved curing processes, and standardisation of block dimensions, have enhanced the compressive strength and durability of sandcrete blocks. Studies by Andohful et al. [16] have shown that standardised blocks with consistent dimensions reduce voids and mortar joints in masonry walls, thereby improving the structural integrity and load-bearing capacity of buildings. These improvements are particularly important in low-rise and medium-rise construction, where block walls often serve as both load-bearing and partition elements. However, these gains in strength and uniformity have come at a cost to worker health, as standardised blocks are often heavier and less ergonomically designed, increasing the physical strain on masons during manual handling [17].

Sandcrete masonry block units are modern construction materials made from sand, cement, and water, providing building comfort [18]. Masonry units comprise almost 90% of homes and facilities worldwide, demonstrating their importance in the building industry [19]. There are different shapes and sizes of blocks. The majority of these blocks are 150 mm wide [20]. According to Onyia et al. [21], the solid and hollow sandcrete blocks have the following dimensions: 450 mm × 225 mm to 450 mm × 150 mm and 450 mm × 113 mm to 450 mm × 100 mm. According to Akorli et al. [22], the typical measurements of sandcrete blocks are 125 mm × 225 mm × 450 mm and 150 mm × 225 mm × 450 mm. The two common sizes of sandcrete blocks are 450 mm × 225 mm × 225 mm and 450 mm × 150 mm × 225 mm rectangular prisms, which can either be hollow or solid [23].

Material handling that involves manual labour is known as manual material handling (MMH). MMH is the manual process of lowering, pushing, pulling, carrying, and elevating building materials and units by OSHA and the OSH Administration [24]. Manual handling refers to tasks requiring the movement, grasping, or restraining of objects, often involving hazardous conditions such as high-force posture, repetitive motion, handling people, unstable loads, and prolonged vibration exposure [8]. These jobs can be automated or labour-intensive, with manual labour performed in various weather conditions [25]. Handling dangerous construction-related manual material causes injuries. Workers may face physical risk factors, fatigue, and accidents due to incorrect or excessive performance of construction-related tasks [17,26,27]. Manual material handling duties pose a major risk to the health of personnel [24].

A lifting task involves moving an object from a starting point to a destination at work [28]. Humans use their spines to lift and handle materials, with object dimensions determining hand distance and orientation, ensuring constant hand distance [28]. According to Lavender et al. [29], sagittal symmetric lifts involve lifting jobs with a specific sequence, starting at a hand height of 270 mm and ending at the elbow level. A study found that when larger loads were raised quickly and over a greater reach distance, participants’ hands were 880 mm off the ground in the mid-frontal plane, with a hand height of 270 mm in the mid-sagittal plane. This results in increased sagittal plane moments, causing a twisted spine, hips, and knees, and is intensified by the rapid lifting [8].

The risk of shoulder injuries is increased by lifting large or heavy weights because they can induce glenohumeral instability and greater activation of the shoulder muscles [30]. The adjustment of shoulder muscles is dependent on the lifting size, amount of weight raised, and work stage on changes in glenohumeral joint stiffness [30]. Musculoskeletal problems are among the most prevalent and dangerous outcomes of manual material handling. Musculoskeletal problems harm workers’ productivity and health [26,27]. Risky manual handling practices can give rise to musculoskeletal illnesses, encompassing soft tissue wounds affecting the neck, legs, arms, shoulders, or wrists, back injuries, hernias, sprains, strains, and joint issues [31]. The two biggest risk factors for low back problems in manual material handlers are load type and weight. The most dangerous tasks seem to be lifting, bending, and twisting; lifting heavy objects increases the frequency and severity of lower back discomfort. There appears to be a substantial risk of low-back pain associated with jobs that include lifting, lowering, pushing, tugging, carrying, and holding goods, as well as abrupt, frequent bending over and bodily motions [24]. The correlation between twisting-intensive lifting jobs and low back discomfort has been established, and these duties may be a risk factor for back injuries sustained in the workplace [28].

Ailments affecting the body’s tendons, nerves, muscles, and supporting structures are referred to as “musculoskeletal disorders” (MSD) [25]. Musculoskeletal diseases (MSDs) in the workplace mostly affect the back, shoulders, hands, hips, knees, feet, wrists, and neck [32]. Construction projects required long work hours and physically demanding tasks such as heavy lifting, labour-intensive manual labour and rigid work postures [33]. Significant biomechanical strains are frequently experienced by construction workers when performing repetitive manual handling tasks such as pushing, tugging, dragging, and lifting. Mechanical spinal loading from manual tasks, including pushing, pulling, lifting, and carrying, has been linked to WMSDs, including low back problems, due to repetitive handling, intense effort, rapid movement, and brief recovery intervals [17,34]. Construction labour is physically and emotionally demanding, leading to strained and injured musculoskeletal systems. Factors such as heat exposure, uneven work schedules, and rugged terrain can cause discomfort in the ligaments, tendons, muscles, bones, and other musculoskeletal elements. This discomfort can be acute or chronic, can be mild to severe, and can be caused by arthritis, overuse, trauma, and poor posture [35].

Bad posture, linked to WMSDs, is one of the eight risk factors in ergonomic evaluation measures. Employment-related postures, like “contributory work” or “effective work,” can affect productivity and provide insight into an individual’s production level. Research shows that workers’ postures can cause harm, with a 98% accuracy rate in emerging industries, and increase the risk of musculoskeletal problems [36]. The construction industry requires a thorough assessment of employees’ posture to enhance safety, health, and productivity [17,37]. The World Health Organisation (WHO) states that various workplace risk factors can lead to various ailments such as cancer, musculoskeletal issues, hearing loss, stress-related ailments, respiratory issues, blood flow issues, and multiple infectious diseases [38].

Workers often face various health issues due to a lack of awareness and prioritisation of their health and working environment, including sunken eyes, stomach issues, fatigue, numbness, bloating, headaches, back, neck, shoulder, palms, wrists, and knee discomfort [39]. WMSDs are prevalent in the construction sector due to the physically demanding nature of work, overexertion, awkward stances, and repetitive movements [37]. Manual material handling accounts for 90% of jobs in the construction sector, and fatigue is a prevalent risk [40]. Back pain annually results in 264 million lost workdays, affecting approximately one in four global workers [36]. In engineering psychology, “fatigue” refers to reduced work capacity, lower job efficiency, and increased workplace incidents or mistakes. Conversely, in the context of occupational safety and health, weariness is defined as a reduction in employees’ physical or mental capacities after extended periods of heavy workload labour [41]. Physical and mental exhaustion are physiological alterations brought on by prolonged workdays and high muscle lactic acid generation. These changes impair mood, irritability, and responsiveness [41]. A summary of the health problems is presented in Table 1.

## 3. Research Methods

### 3.1. Research Approach

This study adopted a qualitative research approach, employing structured questionnaires and field measurements to assess the musculoskeletal health effects of manual sandcrete block handling among construction artisans, specifically masons. The choice to opt for any specific strategy, according to Naoum [42], is based on the purpose of the study and the kind and accessibility of information for the study. In line with that, the study adopts a qualitative approach to accomplish the study’s objectives.

**Figure 1:**
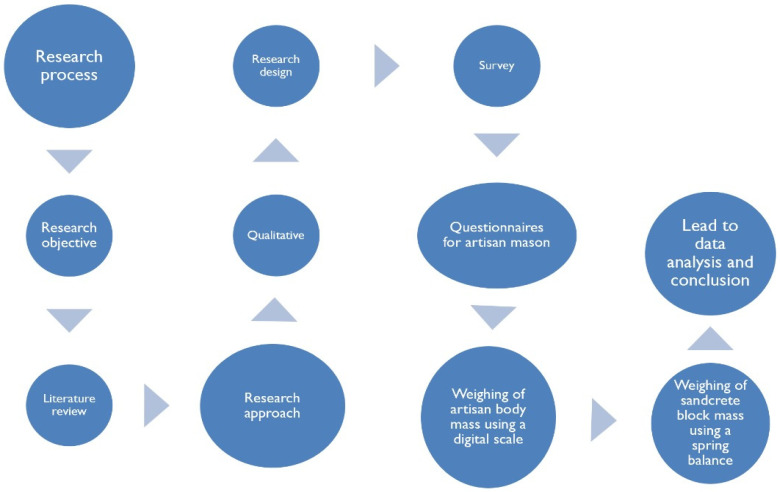
Research Process. Source: Author’s construct (2025).

The approach enabled the collection of both subjective and objective data to evaluate the physical health status of artisans and the ergonomic implications of block handling at construction sites as shown in the research process in Figure 1.

### 3.2. Data Collection Instruments and Procedure

Data were collected through a structured questionnaire administered to artisans working on construction sites in the Oforikrom and Ejisu municipalities in the Ashanti Region of Ghana. The questionnaire was designed in English and translated into local languages to ensure comprehension and accurate responses. Trained field assistants assisted in administering the questionnaires and explaining items where necessary.

The questionnaire was divided into three main sections:

1. Demographic Information: This section gathered data on their gender, qualifications, years of experience, nature of work, and work hours.
2. Work Practices and Manual Handling Activities: This section included questions on how often artisans lift sandcrete blocks, typical postures used, lifting techniques, and work durations.
3. Musculoskeletal Health Symptoms: This section used closed-ended Likert-scale items to capture the presence and severity of musculoskeletal symptoms (e.g., lower back pain, joint pain, muscle fatigue) and how these symptoms impact work life. A 5-point Likert scale was used: 1 = Strongly Agree; 2 = Agree; 3 = Neutral; 4 = Disagree and 5 = Strongly Disagree.

A total of 425 questionnaires were distributed, out of which 402 were completed and returned, yielding a response rate of 95%. Respondents were selected through a purposive sampling technique, based on the following inclusion criteria:

1. Primary engagement in walling/blockwork using sandcrete units;
2. A minimum of ten years of experience in the construction industry.

This approach was chosen to ensure that the study focused on individuals with substantial exposure to manual sandcrete block handling, thereby enhancing the validity of the findings.

In addition to the questionnaire, field measurements were taken to obtain objective data. The body masses of the artisans were measured using a digital body mass scale, and the weights of the sandcrete blocks were recorded using a spring balance. Blocks were hung and weighed on-site to reflect real-time conditions and load burdens typically encountered by workers.

### 3.3. Study Area and Population

The study targeted artisan masons in Oforikrom and Ejisu, within the Ashanti Region of Ghana. These locations were selected due to their high level of informal construction activities. As noted by Fobiri et al. [43], the exact number of masons in these communities is undefined, making the use of purposive sampling suitable for accessing knowledgeable participants.

According to Pandey and Pandey [44], a population refers to the characteristics of a specific group from which a sample is drawn. In this study, the population included all experienced masons involved in manual sandcrete block handling in the selected districts.

### 3.4. Data Analysis

Data from the questionnaires were coded and entered into SPSS software version 25 for analysis. The following data analysis methods were used:

- Descriptive statistics (frequencies, percentages, means, and standard deviations) were used to summarise demographic data and responses related to work practices and health symptoms.
- Mean Item Scores were calculated to determine the severity and frequency of musculoskeletal symptoms.
- Pearson’s Correlation Coefficient was used to test the relationship between variables such as years of experience, frequency of block lifting, and reported musculoskeletal symptoms.
- Standard deviation was used to assess variability in responses and body mass measurements.

This analytical approach provided a robust understanding of the association between manual handling practices and health outcomes.

### 3.5. Ethical Considerations

The study involved human participants and adhered strictly to ethical research standards. Informed consent was obtained from all participants before data collection. Participants were assured of the confidentiality of their responses, and all data were anonymised. Participation was voluntary, and individuals were informed of their right to withdraw at any point without consequence.

## 4. Results

### 4.1. Background Characteristics

Table 2 presents the study’s background information. Educational levels, years of work experience, and working conditions related to sandcrete blocks were analysed. The distribution of these variables was examined using frequencies and percentages.

From Table 2 above, 10% of the respondents were junior high school graduates, and 50% of the respondents completed basic education. Based on working experience, 16 to 20 years represent (53.5%) of working experience and 10 to 15 years represent (44.8%) of working experience. It can be deduced that a majority have 16 to 20 years of working experience. All the artisans in the survey area were exclusively male (100%). The respondents possess experience in providing information in the study area.

### 4.2. Working Conditions of the Artisan Mason

The data in Table 3 show that artisan masons had an average body weight of 61.53 kg (SD = 7.55). They handled 125 mm blocks weighing 25.39 kg (SD = 0.82) and 150 mm blocks weighing 30.29 kg (SD = 0.77). On average, masons laid 106.84 (±20.22) 125 mm blocks and 82.65 (±10.27) 150 mm blocks per day. The average working hours per day were 7.7 h (SD = 0.62), with most masons (74.9%) resting for 1 h and 25.1% resting for 30 min. Regarding weekly schedules, 77.9% of artisans worked six days, 17.7% worked five days, and 4.5% worked seven days. These results indicate high daily workloads, frequent repetitive lifting, and limited rest periods among the artisans.

### 4.3. Descriptive Statistics of the Problems Faced by the Artisans

This section presents the descriptive statistics of the variables in the constructs: general problems, lifting, pulling and pushing, bending and twisting, falling, and weather conditions. The mean scores with standard deviations were estimated, and the results were ranked based on the mean scores from the highest agreement level to the lowest agreement level under each construct.

Table 4 shows descriptive statistics of masons’ work-related health risks. Among general problems, hernia (Mean = 4.06) and muscle strains (Mean = 3.98) were the most frequently reported. For lifting tasks, low-back pain (Mean = 4.74) and joint pain (Mean = 4.71) ranked highest. Pulling and pushing activities most commonly resulted in chronic pain (Mean = 4.28) and numbness (Mean = 4.28), while bending and twisting were primarily associated with muscle pain (Mean = 4.26) and low-back pain (Mean = 4.11). Falling objects led to the highest incidence of bruises (Mean = 4.46) and chronic pain (Mean = 4.22). Weather conditions were most linked to headaches (Mean = 4.11) and muscle pain (Mean = 3.15). Overall, the results indicate that masons experienced multiple musculoskeletal and injury-related risks across different work activities, with varying severity depending on task type.

### 4.4. Relationship Between Background Characteristics and the Working Problem

The relationship between the background characteristics (highest educational level, years of working experience, respondents’ weight, the weight of blocks (125 mm and 150 mm), working hours, and the number of working days in the week. In addition, these variables were correlated with the masons’ problems associated with general problems, such as lifting, pulling and pushing, bending and twisting, falling, and weather conditions. The results in Table 5 and Table 6 show the correlation between the coefficient and *p*-values.

Table 5 presents the correlation analysis between respondents’ weight and working conditions. Artisan weight was significantly negatively correlated with the weight of 125 mm blocks (r = −0.202, *p* < 0.01) and 150 mm blocks (r = −0.248, *p* < 0.01), indicating that heavier masons tended to handle lighter blocks. There was also a negative correlation between artisan weight and the number of blocks laid per day for both 125 mm (r = −0.115, *p* < 0.05) and 150 mm blocks (r = −0.166, *p* < 0.01), as well as with daily working hours (r = −0.133, *p* < 0.01) and resting hours (r = −0.217, *p* < 0.01). Significant positive correlations were observed between block weights, number of workers, resting hours, and working days (*p* < 0.01), suggesting interrelationships among workload, team size, and schedule. These results indicate that masons’ body weight and work conditions are significantly associated with both workload and rest patterns.

N = 402; NRHD = number of resting hours per day; NWDW = number of working days per week.

### 4.5. Relationship Between Background Characteristics and Working Problems

Table 6 shows the correlations between background characteristics and working problems among artisan masons. The highest qualification was significantly positively correlated with lifting (r = 0.259, *p* < 0.01) and negatively correlated with weather-related problems (r = −0.154, *p* < 0.01). Years of working experience showed significant positive correlations with general problems (r = 0.099, *p* < 0.05) and lifting (r = 0.245, *p* < 0.01), and a negative correlation with weather conditions (r = −0.123, *p* < 0.05). Respondents’ weight (RWeight) was positively associated with weather conditions (r = 0.129, *p* < 0.05) but not significantly with other working problems. The weight of 150 mm blocks was positively correlated with lifting (r = 0.207, *p* < 0.01) and pulling/pushing (r = 0.103, *p* < 0.05), and negatively correlated with weather conditions (r = −0.199, *p* < 0.01). The number of workers was significantly associated with lifting (r = 0.346, *p* < 0.01) and weather conditions (r = −0.104, *p* < 0.05). Number of days worked per week showed positive correlations with lifting (r = 0.270, *p* < 0.01), pulling/pushing (r = 0.114, *p* < 0.05), and falling (r = 0.125, *p* < 0.05), and a negative correlation with weather conditions (r = −0.113, *p* < 0.05). These results indicate that education, experience, workload, and work schedule are significantly associated with specific working problems among masons (N = 402; ** *p* < 0.01; * *p* < 0.05).

## 5. Discussion of Results

### 5.1. Discussion on Background Characteristics

Table 2 presents the demographic profile of the respondents. All participants were male (100%), reflecting the gendered nature of masonry work in Ghana, where sandcrete block handling and related construction tasks are predominantly performed by men. Regarding educational background, 39.8% of the respondents had no formal education, 50.2% had basic education, and 10.0% attained junior high-level education. This indicates that most artisan masons possess only basic literacy and technical skills, which may influence their understanding and application of occupational safety and ergonomic practices.

In terms of work experience, the majority of respondents (53.5%) had 16–20 years of experience, followed by 44.8% with 10–15 years, while only 1.7% had worked for more than 20 years. This suggests that most masons are experienced workers who have spent many years in the trade, potentially increasing their exposure to repetitive manual tasks and the risk of developing musculoskeletal problems over time. Overall, the respondents represent a mature and highly experienced but low-educated male workforce, characteristic of Ghana’s informal construction sector.

### 5.2. Discussion on the Working Conditions of Artisans

Table 3 presents essential data on the working conditions of artisan masons engaged in manual sandcrete block handling. The average body weight of the masons was 61.53 kg (SD = 7.55 kg), which places most workers within a moderate weight range. This is a significant observation when evaluated alongside the average weight of the sandcrete blocks handled daily. According to the National Institute for Occupational Safety and Health (NIOSH) lifting equation, the recommended weight limit for safe manual lifting under ideal conditions is approximately 23 kg [45]. In this study, the 125 mm blocks averaged 25.39 kg (SD = 0.82 kg), and the 150 mm blocks averaged 30.29 kg (SD = 0.77 kg), both exceeding NIOSH’s safe lifting threshold. This suggests that even under optimal conditions, the loads being lifted surpass ergonomic safety limits, posing a clear risk of musculoskeletal strain, particularly in the lower back.

The average number of blocks laid per day, 106.84 (±20.22) for 125 mm and 82.65 (±10.27) for 150 mm blocks, implies repeated heavy lifting throughout the day. Research by Coenen et al. [46] and Soares et al. [47] has established that repetitive manual lifting of heavy objects is a major risk factor for cumulative trauma disorders, particularly low back pain (LBP), which has also been widely reported among construction workers. These findings align with those of Ryu et al. [11], who demonstrated that poor posture combined with repetitive lifting increased biomechanical load on spinal structures.

Furthermore, the daily workload reported an average of 7.7 working hours, with only 30 to 60 min of rest, highlighting inadequate recovery periods. Prolonged static and dynamic loading without sufficient rest has been shown in occupational health literature to reduce muscle recovery and increase fatigue [48]. This work-rest pattern contributes to the chronic nature of musculoskeletal complaints, which, over time, may lead to long-term disability or reduced functional capacity.

The work schedule, with 77.9% of artisans working six days a week, mirrors findings in similar low-resource construction contexts where high physical demands are paired with low ergonomic support. Tao et al. [12] found that inadequate recovery between workdays, especially among informal construction workers, leads to persistent musculoskeletal symptoms. In addition, long working hours and high-frequency lifting have been associated with increased inflammatory responses and microtrauma in muscles and tendons [49].

The results confirm that artisan masons in Ghana are subject to occupational exposures, including excessive load weights, high repetition, limited rest, and extended work weeks, all of which are well-documented contributors to musculoskeletal disorders (MSDs). Despite these known risks, the informal and labour-intensive nature of the Ghanaian construction industry often means that ergonomic considerations are neglected, and there is limited implementation of preventive measures. These findings highlight the urgent need for training in safe lifting techniques, ergonomically informed work practices, and policy enforcement in the Ghanaian construction sector to mitigate long-term health consequences.

### 5.3. Discussion on the Health Problems Faced by Artisans

The results showed that the main problems associated with general masons’ work included hernias, muscle strains, headaches, sprains, waist pain, and low-back pain, as presented in Table 4. These outcomes are consistent with findings from Soares et al. [47], who noted that manual labour involving repetitive lifting, bending, and awkward postures significantly contributes to musculoskeletal disorders (MSDs), particularly in occupations like construction, where such tasks are frequent and intense.

Under the problems associated with lifting objects, the most commonly reported issues included low-back pain, joint pain, waist pain, muscle pain, bruises, abdominal pain, and hernia. These complaints are biomechanically linked to spinal compression and muscular overload, particularly when lifting heavy loads without mechanical assistance or proper technique. According to Marras et al. [50], lifting tasks that involve trunk flexion and rotation significantly increase the risk of lumbar spine injuries, especially when performed repeatedly, as observed in this study. The high prevalence of low-back pain supports existing literature that identifies the lower back as one of the most vulnerable anatomical sites in manual handling occupations.

Concerning pulling and pushing tasks, the commonly reported issues were chronic pain, numbness, muscle pain, cuts, joint pain, bruises, hernia, and headache. These are consistent with evidence from Punnett and Wegman [49], who found that pushing and pulling heavy loads, particularly on uneven or unstable surfaces, contributes to repetitive strain injuries, nerve compression, and joint degeneration. The presence of numbness and chronic pain suggests possible nerve involvement, such as compression syndromes or prolonged postural loading of the upper limbs and lower back.

For bending and twisting, the mean scores ranged from 4.26 to 3.49, indicating general agreement among respondents about the frequency of associated health problems. The dominant complaints muscle pain, low-back pain, muscle strain, bruises, abdominal pain, chronic pain, and hernia, are expected outcomes of dynamic spinal loading, particularly under asymmetrical postures. Research by Ryu et al. [11] and Tao et al. [12] similarly demonstrated that repeated trunk flexion and torsion, especially under load, place significant stress on the intervertebral discs and spinal muscles, increasing the risk of injury.

Problems associated with falling objects included bruises, sprains, chronic pain, small fractures, numbness, abdominal pain, and headaches, with mean scores ranging from 4.46 to 3.87, also indicating a high level of agreement. This is consistent with the findings of Kudlinski [51], who identified struck-by-object injuries as a major hazard in construction work, often caused by poor material handling, lack of protective gear, and unstructured work environments. The presence of small fractures and sprains may reflect acute trauma, while headaches and abdominal pain could indicate both physical strain and secondary stress responses.

The influence of weather conditions associated with headaches, bruises, muscle pain, low-back pain, sprains, and chronic pain is also significant. Prolonged exposure to extreme heat or humid working environments, common in Ghana, exacerbates fatigue, reduces focus, and can increase the likelihood of slips and muscle exhaustion. According to the World Health Organisation (WHO) [6], climatic conditions can influence the physiological workload on labourers, especially when hydration, rest, and protective gear are insufficient factors often overlooked in informal construction settings.

Overall, the results reveal a multifactorial burden of musculoskeletal health issues, with causes spanning biomechanical stress, environmental exposure, and unsafe work practices. These findings are in alignment with the global literature on occupational ergonomics, which stresses that manual material handling without ergonomic intervention leads to both acute injuries and chronic degenerative conditions [52]. The consistency between the findings and existing research underscores the urgency of incorporating ergonomics, worker training, and regulatory oversight into industry practice, especially within labour-intensive construction sectors like Ghana’s.

### 5.4. Discussion on the Relationship Between Background Characteristics and Health Problems

The study found a significant negative relationship between respondents’ body weight and the weight of the sandcrete blocks they handled. Specifically, the correlation between artisan weight and the 125 mm block was −0.202 (*p =* 0.000), and for the 150 mm block, −0.248 (*p =* 0.000), suggesting that heavier masons tended to handle lighter blocks and Vice Versa. This result is intriguing, as it contradicts common assumptions that heavier or physically larger individuals are naturally assigned to heavier manual tasks. It could be influenced by site-specific practices such as task rotation or block availability, or by the use of a single block type at a time, limiting the mason’s ability to choose.

These findings can also be viewed through the lens of workload distribution and self-selection bias. According to DiDomenico & Nussbaum [53], Perry et al. [54] and MacDonald [55], workers may subconsciously adjust their workload based on their perceived physical capacity. In high-risk manual tasks, heavier individuals may either self-select into less physically taxing roles to avoid fatigue or be assigned lighter blocks by supervisors aware of health risks. Additionally, Ryu et al. [11] suggest that the recommended maximum cumulative weight for lifting per day is 3300 kg. In this study, the calculated total daily lifted weight was 2717 kg for 125 mm blocks and 2505 kg for 150 mm blocks, which both fall below this threshold. Thus, while block weight alone may seem high, the total daily lifting volume remains within ergonomic safety limits, supporting the threshold proposed by Ryu et al. [11].

The negative relationship between the artisans’ weight and the number of blocks laid per day also highlights how heavier masons may pace their work more slowly, possibly due to reduced mobility or fatigue thresholds. This is supported by the observed negative correlation between weight and working hours per day (coefficient = −0.133, *p* < 0.05). This suggests that heavier workers may experience an earlier onset of fatigue, reducing their ability to sustain long work durations. Literature by Kermavnar et al. [56] supports this, noting that increased body mass may be associated with higher metabolic strain and increased risk of early fatigue during physically demanding tasks.

Moreover, the negative correlation between body weight and resting hours per day (coefficient = −0.217, *p* < 0.05) could reflect reduced resilience or increased perceived effort among heavier artisans, resulting in the need for more frequent or longer breaks. This contrasts with the actual findings, which showed heavier artisans taking fewer rest breaks, possibly due to economic pressures or poor awareness of occupational health needs. According to Punnett and Wegman [49], insufficient rest during repetitive tasks increases the likelihood of cumulative musculoskeletal injuries, especially in jobs involving manual lifting and prolonged static postures.

Taken together, these results reflect complex interactions between body mass, work allocation, and task endurance. While block weights remain within Ryu et al.’s [11] recommended limits, the frequency and duration of lifting, especially among lighter masons lifting heavier blocks, pose a risk of overexertion injuries. Additionally, the variability in rest periods, regardless of weight, suggests poor implementation of ergonomic rest-work cycles, which are recommended in ISO 11228 standards for manual handling tasks [57].

These correlations reinforce the importance of tailoring work tasks not only to the average physical capacity of workers but also to individual ergonomic thresholds. Without such considerations, both lighter and heavier workers may be at risk of injury, the former due to overexertion, and the latter due to fatigue and reduced endurance. Future interventions should consider body mass index (BMI), lifting capacity, and daily workload to better allocate tasks and structure rest schedules, particularly in informal construction settings.

The relationship between respondents’ highest educational qualification and health problems revealed a significant positive correlation with lifting-related issues (coefficient = 0.259, *p =* 0.000 < 0.05), indicating that artisans with higher education levels reported more problems related to lifting blocks and associated materials. While this finding may appear counterintuitive, it could be influenced by a greater awareness and self-reporting of health issues among more educated workers. According to Lowe et al. [58], workers with higher educational attainment are more likely to recognise and report musculoskeletal symptoms, possibly due to better health literacy and greater concern for personal well-being. It is also possible that such workers may not be as physically conditioned as their less-educated counterparts, who have spent more years performing strenuous manual labour.

In contrast, there was a significant negative relationship between educational qualification and weather-related health problems (coefficient = −0.154, *p =* 0.002 < 0.05), suggesting that artisans with higher education may be more adept at managing environmental challenges. This could stem from their increased knowledge of protective strategies or preference for roles that are less exposed to extreme weather, as highlighted in WHO’s occupational health recommendations [6].

Interestingly, there was no significant relationship between education level and other general problems, such as pulling, pushing, bending, twisting, or falling. This may indicate that exposure to physical risks is relatively uniform across educational levels in informal construction settings, where task allocation is based more on labour availability than educational background.

Regarding years of experience, a positive correlation was found with lifting-related problems (coefficient = 0.245, *p =* 0.000 < 0.05), suggesting that cumulative exposure to manual lifting over time contributes to musculoskeletal deterioration. This aligns with the findings of Punnett and Wegman [49], who observed that the length of time in physically demanding jobs is directly associated with the development of chronic musculoskeletal disorders, particularly in the lower back and shoulders. The wear and tear from repetitive lifting likely builds up over time, affecting even experienced masons who may have adapted their technique but are still exposed to high physical loads.

Conversely, there was a significant negative association between years of working experience and general musculoskeletal problems, implying that more experienced workers may adopt coping strategies or efficient techniques over time to minimise injuries during pushing, pulling, bending, and twisting tasks. This is consistent with research by Odebiyi and Okafor [59] and Gallagher and Heberger [60], who found that skill development in manual labour tasks can lead to better load distribution, reduced effort, and decreased injury rates in certain repetitive motions.

The number of working hours per day was positively associated with lifting problems (coefficient = 0.346, *p* < 0.05), suggesting that longer work durations increase physical strain. This is supported by da Costa and Vieira [52], who emphasised that extended shifts without adequate rest lead to muscular fatigue and increased susceptibility to injury. The finding of a negative relationship between hours worked and weather-related health issues implies that longer hours may reduce exposure to adverse weather, potentially due to early start times or work in partially sheltered environments. However, this may also mask underlying climatic stress, particularly if rest breaks and hydration are inadequate.

The analysis further revealed that the number of working days per week was positively correlated with lifting problems (coefficient = 0.270, *p* < 0.05), as well as with pushing and pulling (coefficient = 0.114, *p =* 0.022), and issues related to falling (coefficient = 0.125, *p =* 0.012). These findings indicate that cumulative work stress increases with frequency of exposure, supporting existing research that links short recovery periods with a higher risk of work-related musculoskeletal disorders (WMSDs) [61]. As the number of workdays increases, the opportunities for physiological recovery decline, compounding fatigue and risk of injury. Surprisingly, the data showed that weather-related problems decreased with increased workdays, which might be attributed to adaptive behaviours or the assignment of less weather-exposed tasks to more frequently available workers.

Taken together, these results highlight the complex interplay between worker characteristics (such as education and experience) and occupational exposures. While certain findings, such as experienced masons reporting fewer general problems, suggest the value of on-the-job learning, others (like higher-educated workers reporting more lifting issues) point to the need for targeted ergonomic interventions that are not solely based on skill or tenure. Instead, policies should consider individual worker profiles, including their background and physical capabilities, in assigning tasks and setting work schedules.

These insights are critical in guiding training programs and policy formulation. For instance, more educated artisans could be better utilised in supervisory or health and safety roles, while experienced but ageing masons could benefit from assistive lifting equipment or task rotation to reduce cumulative strain. Additionally, ensuring structured work-rest cycles and limiting consecutive workdays can help mitigate both environmental and physical health risks.

The study’s findings have key implications for construction practice, policy, and future research. Practically, there is a need for ergonomic interventions such as mechanical lifting aids, better task rotation, and adequate rest to reduce physical strain on masons. Task assignments should also consider workers’ physical capacities, especially for those with higher body weight or experience. On a policy level, introducing maximum block weight standards and reducing import taxes on construction machinery could promote safer practices and mechanisation. Training institutions should integrate occupational health and safety into artisan education. For future research, longitudinal and biomechanical studies are recommended to track health outcomes and validate physical strain. Comparative and intervention-based research could further inform the development of safer, more efficient construction practices.

## 6. Conclusions

This research aimed to determine the effect of the mass of standardised sandcrete blocks on the musculoskeletal health of construction artisans who manually handle them. The findings revealed a high prevalence of musculoskeletal disorders, including low back pain, muscle strain, joint pain, and chronic fatigue, among artisan masons. These outcomes are largely attributable to repetitive manual lifting, bending, and awkward postures during blocklaying activities. The average block weight (25.39 kg for 125 mm and 30.29 kg for 150 mm) was found to impose a significant physical strain on workers, especially given the high number of blocks laid daily (over 100), and the long working hours (averaging 7.7 h per day), often with minimal rest.

Importantly, this study contributes new empirical data to the body of knowledge on ergonomics and occupational health in the construction industry in low- and middle-income countries, particularly Ghana. While existing studies have highlighted the general dangers of construction work, limited attention has been paid to the specific physical burden posed by standardised block weights and the relationship between worker characteristics such as body mass, education, experience and health outcomes. This study fills that gap by providing statistical correlations between worker attributes and musculoskeletal risks, offering insights that can inform more tailored ergonomic interventions in the industry.

The study also underscores the influence of contextual factors such as low formal education, which may limit artisans’ awareness of injury prevention techniques, and the culture of extended work schedules with insufficient rest. These findings highlight a crucial need for industry stakeholders, including construction firms, block manufacturers, and technical training institutions, to implement targeted ergonomic interventions. These may include the production of lighter-weight blocks, the use of mechanical lifting aids, and mandatory training in safe lifting techniques and posture. Promoting awareness of the risks of musculoskeletal disorders through structured health and safety education is also vital.

Practically, this research supports the integration of ergonomics into artisanal training programs and calls for policy reforms that prioritise worker health in informal construction sectors. It provides evidence-based justification for shifting from purely manual to semi-mechanised construction practices, particularly in regions where labour-intensive methods remain dominant.

However, the study is limited by its focus on masons and sandcrete blockwork only, without examining other artisanal roles or materials within the construction industry. Additionally, the use of a predominantly quantitative approach may not fully capture the lived experiences or coping mechanisms employed by artisans. The block sizes and demographic characteristics in the selected regions may also limit generalisability to other contexts.

Future research should employ regression analysis to model the relationship between artisans’ demographic and work-related factors, such as age, educational qualification, gender, artisan weight, block weight, and the number of blocks laid per day, and their musculoskeletal health conditions. This approach would provide a more detailed understanding of how these variables jointly influence the occurrence and severity of work-related health problems, allowing for predictive insights and targeted ergonomic interventions within the construction industry.

In conclusion, this study makes a valuable contribution to the discourse on construction worker health by quantifying the ergonomic risks associated with sandcrete block handling and offering practical pathways for intervention. It bridges an important gap between occupational health research and on-ground construction practices in developing contexts.

## Figures and Tables

**Table 1:** Health risks from sandcrete block usage and their causes.

S/No	Health Risks	Causes					
		Lifting of Blocks	Fall of Blocks	Pushing and Pulling of Blocks	Bending and Twisting	Weather Conditions	References
1	Abdominal pain	x		x	x		[17,32,33]
2	Headache	x		x		x	[30]
3	Numbness	x		x			[12]
4	Joint pain	x		x	x		[24]
5	Waist pain	x		x	x		[34]
6	Muscles pain				x		[34]
7	Low-back pain	x		x	x		[35]
8	Chronic pain	x		x	x		[36]
9	Hernias	x		x	x		[37]
10	Cuts		x				[38]
11	Bruises	x	x				[39]
12	Sprains	x	x	x	x		[40]
13	Tears	x	x	x			[41]
14	Small fractures	x	x	x			[38,39]
15	Muscle strains	x	x	x	x		[37,38]

Source: Literature review, 2024.

**Table 2:** Respondent profile.

Background Characteristics	Frequency	Percent
Gender		
Male	402	100.00
Female	0	0.00
Total	402	100.0
Highest qualification	-	
No formal education	160	39.8
Basic education level	202	50.2
Junior high-level	40	10.0
Total	402	100.0
Years of working experience	**-**	
10–15 years	180	44.8
16–20 years	215	53.5
21–25 years	4	1.0
26 years and above	3	0.7
Total	402	100.0

Source: Field data, 2024.

**Table 3:** Working conditions of the artisan mason.

Working Conditions	Mean	Std. Deviation
Weight of the artisan Mason	61.53	7.55
Weight of the 125 mm block	25.39	0.82
Weight of the 150 mm block	30.29	0.77
The number of 125 mm blocks you laid in a day	106.84	20.22
The number of 150 mm blocks you laid in a day	82.65	10.27
The number of hours you work in a day	7.70	0.62
	**Frequency**	**Percent**
The number of resting hours per day	-	
30 min	101	25.1
1 h	301	74.9
Total	402	100.0
Number of working days in the week	-	
5 days	71	17.7
6 days	313	77.9
7 days	18	4.5
Total	402	100.0

Source: Field data, 2024.

**Table 4:** Descriptive statistics of the masons’ work health risks.

Problems	General Problem		Lifting		Pulling and Pushing		Bending and Twisting		Falling		Weather Conditions	
	Mean (SD)	Rank	Mean (SD)	Rank	Mean (SD)	Rank	Mean (SD)	Rank	Mean (SD)	Rank	Mean (SD)	Rank
Abdominal pain	3.73 (1.111)	9	3.89 (0.972)	6	3.93 (1.058)	9	4.02 (0.894)	5	4.02 (0.967)	6	3.00 (1.521)	10
Headache	3.96 (0.993)	3	3.24 (0.680)	10	3.94 (0.869)	8	3.72 (0.783)	12	3.99 (0.927)	7	4.11 (0.990)	1
Numbness (shaking)	3.75 (0.978)	8	3.15 (0.839)	12	4.28 (0.894)	2	3.75 (0.823)	10	4.05 (0.946)	5	2.97 (1.495)	11
Joint Pain	3.65 (1.044)	13	4.71 (0.674)	2	4.00 (0.862)	5	3.49 (0.883)	14	3.87 (0.868)	14	2.90 (1.476)	14
Waist Pain	3.91 (1.045)	5	4.71 (0.670)	3	3.61 (0.795)	13	3.70 (0.824)	13	3.88 (0.943)	13	3.01 (1.524)	9
Muscles Pain	3.69 (0.915)	12	4.62 (0.652)	4	3.60 (0.940)	14	4.26 (0.821)	1	3.95 (0.858)	9	3.15 (1.581)	3
Low-Back Pain	3.88 (0.993)	6	4.74 (0.639)	1	3.77 (0.877)	10	4.11 (0.854)	2	3.91 (0.877)	11	3.09 (1.550)	4
Chronic Pain	3.73 (0.962)	10	2.46 (1.071)	14	4.28 (0.964)	1	4.02 (0.749)	6	4.22 (0.926)	3	3.06 (1.510)	7
Hernia	4.06 (1.002)	1	3.71 (1.155)	7	3.94 (0.960)	7	3.88 (1.121)	7	3.89 (0.854)	12	2.95 (1.524)	13
Muscles Strains	3.98 (0.943)	2	3.66 (1.113)	8	4.13 (0.927)	3	4.06 (1.025)	3	3.93 (0.865)	10	2.97 (1.501)	12
Cut	3.73 (0.917)	11	3.17 (0.922)	11	4.03 (1.011)	4	3.85 (0.942)	8	3.96 (0.937)	8	3.03 (1.545)	8
Sprain	3.94 (0.877)	4	3.26 (0.860)	9	3.74 (0.932)	11	3.80 (0.978)	9	4.25 (0.811)	2	3.09 (1.578)	5
Bruises (scratches)	3.81 (0.816)	7	3.91 (0.747)	5	3.99 (1.046)	6	4.03 (1.011)	4	4.46 (0.850)	1	3.16 (1.619)	2
Small Fractures	3.64 (0.954)	14	2.59 (1.185)	13	3.66 (0.809)	12	3.74 (0.932)	11	4.15 (0.839)	4	3.07 (1.547)	6

Source: fieldwork, 2024.

**Table 5:** Correlations Analysis of Respondents’ Weight and Working Conditions.

		RWeight	Weight 125 mm Blocks	Weight 150 mm Blocks	No. 125 mm Blocks	No. 150 mm Blocks	Workers	NRHD	NWDW
RWeight	Coeff.	1							
	*p*-value								
Weight 125 mm blocks	Coeff.	−0.202 **	1						
	*p*-value	0.000							
Weight 150 mm blocks	Coeff.	−0.248 **	0.919 **	1					
	*p*-value	0.000	0.000						
No. 125 mm blocks	Coeff.	−0.115 *	−0.178 **	−0.208 **	1				
	*p*-value	0.021	0.000	0.000					
No. 150 mm blocks	Coeff.	−0.166 **	0.151 **	0.148 **	0.331 **	1			
	*p*-value	0.001	0.002	0.003	0.000				
Workers	Coeff.	−0.133 **	0.709 **	0.628 **	−0.174 **	0.093	1		
	*p*-value	0.008	0.000	0.000	0.000	0.063			
NRHD	Coeff.	−0.217 **	0.644 **	0.489 **	−0.111 *	−0.004	0.881 **	1	
	*p*-value	0.000	0.000	0.000	0.027	0.934	0.000		
NWDW	Coeff.	−0.070	0.722 **	0.626 **	−0.183 **	0.051	0.796 **	0.732 **	1
	*p*-value	0.162	0.000	0.000	0.000	0.306	0.000	0.000	
	N	402	402	402	402	402	402	402	402

**. Correlation is significant at the 0.01 level. *. Correlation is significant at the 0.05 level; NRHD: The number of resting hours per day; NWDW: Number of working days in the week.

**Table 6:** Relationship between background characteristics and working problems.

Relationship Between Background Characteristics and Working Problems	Pearson Correlation	*p*-Value	95% CI	
			Lower	Upper
Highest qualification—General problem	0.092	0.064	−0.005	0.189
Highest qualification—Lifting	0.259	0.000 **	0.165	0.348
Highest qualification—Pulling and Pushing	0.083	0.097	−0.015	0.179
Highest qualification—Bending and Twisting	0.067	0.178	−0.031	0.164
Highest qualification—Falling	0.005	0.923	−0.093	0.103
Highest qualification—Weather Conditions	−0.154	0.002 **	−0.248	−0.057
Years of working experience—General problem	0.099	0.047 *	0.001	0.195
Years of working experience—Lifting	0.245	0.000 **	0.151	0.335
Years of working experience—Pulling and Pushing	0.041	0.413	−0.057	0.138
Years of working experience—Bending and Twisting	0.021	0.678	−0.077	0.118
Years of working experience—Falling	−0.012	0.818	−0.109	0.086
Years of working experience—Weather Conditions	−0.123	0.013 *	−0.219	−0.026
RWeight—General problem	−0.015	0.768	−0.112	0.083
RWeight—Lifting	−0.077	0.124	−0.173	0.021
RWeight—Pulling and Pushing	−0.073	0.146	−0.169	0.025
RWeight—Bending and Twisting	−0.084	0.093	−0.180	0.014
RWeight—Falling	−0.010	0.847	−0.107	0.088
RWeight—Weather Conditions	0.129	0.010 *	0.031	0.224
Weight 150 mm block—General problem	0.063	0.209	−0.035	0.160
Weight 150 mm block—Lifting	0.207	0.000 **	0.112	0.299
Weight 150 mm block—Pulling and Pushing	0.103	0.039 *	0.005	0.199
Weight 150 mm block—Bending and Twisting	0.073	0.142	−0.025	0.170
Weigh 150 mm block—Falling	0.019	0.709	−0.079	0.116
Weight 150 mm block—Weather Conditions	−0.199	0.000 **	−0.291	−0.103
Workers—General problem	0.048	0.335	−0.050	0.145
Workers—Lifting	0.346	0.000 **	0.257	0.429
Workers—Pulling and Pushing	0.079	0.116	−0.019	0.175
Workers—Bending and Twisting	0.063	0.211	−0.035	0.159
Workers—Falling	0.095	0.056	−0.003	0.191
Workers—Weather Conditions	−0.104	0.036 *	−0.200	−0.007
Number of days of work in the week—General problem	0.071	0.157	−0.027	0.167
Number of days of work in the week—Lifting	0.270	0.000 **	0.177	0.359
Number of days of work in the week—Pulling and Pushing	0.114	0.022 *	0.017	0.210
Number of days of work in the week—Bending and Twisting	0.070	0.164	−0.028	0.166
Number of days of work in the week—Falling	0.125	0.012 *	0.028	0.220
Number of days of work in the week—Weather Conditions	−0.113	0.023 *	−0.208	−0.015

Source: Fieldwork, 2024. **. Correlation is significant at the 0.01 level. *. Correlation is significant at the 0.05 level.

## Data Availability

The data presented in this study are within this article; however, further information can be sought upon reasonable request to the corresponding author.
